# Detection of SHV type Extended-Spectrum B-lactamase and Risk Factors in *Pseudomonas aeruginosa* Clinical Isolates

**DOI:** 10.12669/pjms.293.3263

**Published:** 2013

**Authors:** Nasrin Bahmani, Rashid Ramazanzadeh

**Affiliations:** 1 Nasrin Bahmani, MS, Microbiology Department, Faculty of Medicine, Kurdistan University of Medical Sciences, Sanandaj-Iran.; 2 Rashid Ramazanzadeh, Associated Professor, Cellular and Molecular Research Center, Faculty of Medicine, Kurdistan University of Medical Sciences, Sanandaj-Iran.

**Keywords:** *P. aeruginosa*, Antimicrobial resistance, Extended-Spectrum beta-Lactamase, SHV

## Abstract

***Objective:*** *Pseudomonas aeruginosa* is one of the most important causes of nosocomial infections and can acquire resistant to many antimicrobials, including β-lactams. The aim of this study was to detect the prevalence of SHV type extended-spectrum beta-lactamase (ESBL), antimicrobial resistance patterns of the *P. aeroginusa* and risk factors in hospitalized patients in two teaching hospitals in Sanandaj, Iran.

***Methodology:*** 123 *P. aeruginosa* were isolated from various clinical specimens. All samples were prepared for double-disk synergy test on the isolates for detection of ESBL. SHV was confirmed by PCR method. Risk factors were evaluated for infection due to *P. aeruginosa*.

***Results:*** The incidence of multiple drug resistance (MDR) in *P. aeroginusa* isolates was 3.85%. The prevalence of ESBL-SHV gene was 10.57%. Days of hospitalization (OR=14.34 CI95% 2.87-25.8), ICU hospitalization (OR=3.4 CI95% 1.24- 9.29), presence of catheter (OR=3.63 CI 95% 1.34-9.84), use of antimicrobials within previous two weeks (OR=5.51 CI95% 1.85-16.43) and use of ventilator (OR=3.7557 CI95%1.29-9) were risk factors for *Pseudomonas *nosocomial infection SHV positive ESBL.

***Conclusion:*** In this study Prevalence of ESBL, SHV gene and MDR in *P. aeroginosa* infection was lower than the prevalence reported from other studies in Iran and this indicated appropriate antimicrobial managements strategies and infection control. In addition, our research data indicate that risk factors such as use of ventilator, use of antimicrobials and ICU hospitalization can be effective in managing *Pseudomonas* infection.

## INTRODUCTION

*Pseudomonas aeruginosa* is one of the most important pathogens causing nosocomial infections including pneumonia, urinary tract infections, and bacteremia; and in recent years has shown multi-drug resistant to many antimicrobials, including b-lactams.^1^ Nowadays antimicrobial resistance is a great problem for the treatment and management of infectious disease. Patients with drug resistance organisms are at risk of negative outcome, even mortality. Designing controlling program will require insights from a range of disciplines including epidemiology, molecular biology and evolutionary biology of resistance genes.^2^

The most prevalent of antimicrobial resistance mechanism in bacterial strains are enzymatic destruction. Among these enzymes, extended spectrum beta-lactamase (ESBL) have been studied extensively. In *P. aeruginosa*, various classes of ESBLs (A, B and D) have been found. Five types of class A ESBLs (PER, VEB, GES and IBC, TEM and SHV) were recently reported.^3^ ESBL productions have been reported from many parts of the world and in this region however, based on our literature survey, there is scanty reports on the isolation of SHV-type ESBLs in *Pseudomonas aeruginosa*.^4^^-^^6^

Multiple drug resistance *P. aeruginosa* (MDR) are defined as isolates resistant to ceftazidime, cefepime, aztreonam, ciprofloxacin, piperacillin, and gentamicin.^7^ Evaluation of the risk factors association Multiple drug resistance *P. aeruginosa* (MDR) are defined as isolates ociated with the acquisition of MDR *P. aeruginosa* has been an area of active research for epidemiologists and physicians around the world.^8^ These factors may provide an improvement in treatment outcome. We survey this study to analysis the risk factors for acquisition of SHV enzyme in *P. aeruginosa* strains isolated from hospitalized patients at two teaching hospitals in Sanandaj, Iran.

## METHODOLOGY

***Study population and specimen types: ***123* P. aeruginosa* strains were isolated from various clinical specimens including (urine 46.34%, wound 7.32%, respiratory tube 30%, blood 9.7%, cerebrospinal fluid 0.81%, and others 1.63%) from patients who were hospitalized in Toohid and Beasat Teaching Hospitals in Sanandaj, Iran.

***Bacterial isolation: ***The bacteria were cultured on MacConkey agar, Kligler iron agar (MAST, UK) and incubated at 37^o^ C in air. *P. aeruginosa* was recognized with positive oxidase test, prepared from production of a pigment on Mueller–Hinton agar (MAST, UK) and grown aerobically in OF (Merck, Germany) medium and by oxidation of glucose. The bacteria were stored at - 20^ o^ C in trypticase soy broth containing 20% glycerol. *P. aeruginosa* ATCC 27853 was used as a positive control.

***Antimicrobial susceptibility testing: ***Antimicrobial susceptibility of the *P. aeruginosa* strains were validated following the Clinical Laboratory Standards Institute (CLSI).^9^ The following antimicrobial agents were tested: amikacin (30 µg), ampicillin (10 µg)**, **cefalotin (30 µg), ceftriaxone (30 µg), ciprofloxacin (5 µg), cotrimoxazole (1.25/23.75 µg), gentamicin (10 µg), tetracycline (30 µg), carbinicillin (100 µg), imipenem (10 µg), nalidixic acid (30 µg), nitroforantoin (300 µg ), norfloxacine (10 µg), cefepim (30 µg), cefdinir (30) ceftizoxime (30 µg), cefotaxime (30 µg) cefotaxime/clavulanic acid (30/10 µg), ceftazidime (30 µg), and ceftazidime/clavulanic acid (30/10 µg).

Phenotypic ESBL screening test for *P. aeruginosa* strain was a combination disc (MAST co). In this method, ceftazidime and cephotaxime alone and in combination with clavulanic acid were used for detection of ESBL production. Difference in inhibition zones for the ceftazidime disc and ceftazidime plus clavulanic acid combination disc of more than ≥ 5 mm indicates the presence of ESBL in the test organism.

***Risk factors: ***Risk factors including nosocomial infection, days of hospitalization, ICU hospitalization, presence of catheter, use of antimicrobials within previous two weeks and use of ventilator, trauma, blood transfusion**, **gender, and age were collected by questionnaire.

***Detection ESBL- SHV by polymerase chain reaction (PCR): ***Polymerase chain reaction (PCR) was performed for all isolates identified as ESBL producers, showing resistance to b-lactams. DNA was extracted by the boiling method.^10^ Template DNA was prepared, a cell pellet from 1.5 ml of overnight culture was resuspended in 500 μl of TE (10 mM Tris, 1 mM EDTA, pH 8.0) after centrifugation and boiling for 10 min for PCR was used as supernatant. Primers and conditions of polymerase chain reaction used in this study were SHV-F 5´- GGGTTATTCTTATTTGTCGC-3.´, and SHV-R 5´- TTAGCGTTGCCAGTGCTC-3.´ PCR condition 94°C 5min, 35 cycles of, 94° C 1min, 58°C 1min, and 72°C 1min.

***Statistical analysis: ***Data were entered into a database using SPSS 11.5 for Windows. Differences between proportions were analyzed using the 2 test. All differences in which the probability of the null hypothesis was p < 0.05 were considered significant.

## RESULTS

During the study period, all of the 123 *P. aeruginosa* were isolated and identified at both hospitals. Table-I shows demographics of patients. The mean age of the patients was 43.39 ± 3.70 yr (Range; 1-82 yr old), and 84 (68.29%) patients were male. Of 123 patients, 18 (14.63%) patients had underlying disease. Of the123 specimens isolated, the most common was urinary infection (57; 46.34%). blood 9.7%, lung 30%, urine 46.34%, wound 7.32%, brain shunt 0.81%, swab 3.25% and the other 1.63% (Table-I). The most resistant antimicrobials tested against *P. aeruginosa* were ceftazidime (23.58%) and cefotaxime (30.48%). The multiple drug resistance (MDR) patterns of the *P. aeroginusa* isolates were 3.85%. Antimicrobial resistance patterns of the SHV gene have showed in Table-II.

Of the 123 *P. aeroginosa* isolates, 22(17.89%) were positive for ESBL production using the double-disc synergy test and 12 (10.57%) were SHV positive.

There was significant relationship between nosocomial infection SHV positive ESBL with, days of hospitalization (OR14.34 CI95% 2.87-25.8), ICU hospitalization (OR 3.4 CI95% 1.24- 9.29), presence of catheter (OR 3.63 CI 95% 1.34-9.84), use of antimicrobials within previous two weeks (OR5.51 CI95% 1.85-16.43) and use of ventilator (OR3.57 CI95% 1.29-9) as shown in Table-III.

## DISCUSSION

In our study 123 specimens *P.aeroginosa* were isolated; blood 9.7%, lung 30%, urine 46.34%, wound 7.32%, brain shunt 0.81%, swab 3.25% and the other 1.63% while the highest specimen was urine , that is similar to other findings reported at a referral center in Iran.^11^

In 2003 from the National Nosocomial Infections Surveillance System regarding intensive care unit (ICU) patients across the USA showed that, *Pseudomonas* spp. were responsible for 18.1% of pneumonias, 3.4% of bloodstream infections, 9.5% of surgical site infections and 16.3% of urinary tract infections.^8^

We surveyed risk factors in *Pseudomonas *nosocomial infection correlation with SHV positive ESBL that days of hospitalization (OR14.34 CI95% 2.87-25.8), ICU hospitalization( OR 3.4CI95% 1.24- 9.29), presence catheter(OR 3.63 CI 95% 1.34-9.84 ), use of antimicrobials within previous two weeks (OR5.51 CI95% 1.85-16.43) and use of ventilator (OR3.57 CI95% 1.29-9.83) were risk factor but there are no significant difference between gender, trauma, mean age and blood transfusion with *pseudomonas* infection. Similarly, to a research in France, there were significant differences between days of hospitalization 0.003, ICU hospitalization 0.014, and presence catheter 0.011with *pseudomonas* infection.^12^ As reported from previous studies use of invasive devices and urinary catheterization were significant^8^^,^^12^ as a risk factor for resistance development. Recently, resistance to antimicrobial agents of *P. aeruginosa* has become a serious problem in clinic and outbreak of MDR phenotype is increasing among *P. aeruginosa* in patients.^13^

In this study, the most of resistant in *P. aeruginosa* to ceftazidime and cefotaxim were (23.58 %), and (30.48%), respectively. These resistance rates are similar in some of studies^11^ and lower than other reports from Iran.^1^^,^^14^^,^^15^ The outbreak of ESBL has previously been described in Iran but the rate of that was different from this study results. In this study frequency of ESBL-producing strains in pseudomonas was 17.89% that lower than to the rates of other studies in Iran^1^^,^^15^^-^^17^ and higher than United Kingdom (3.7%).^18^

In the present study, reported prevalence of SHV gene 10.57%. In Turkey, prevalence SHV production by *Klebsiella pneumoniae*, *Escherichia coli*, *Acinetobacter baumannii* and *Pseudomonas aeruginosa* was 21.87% has been reported^19^ and in Iran was reported by shacheraghi 22% in *P. aeruginosa*^15^ and 26% for *klebsiella pneumonia* by Nasehi^20^ which may reflect these differences in infection control policies. The rates of these enzymes in our study are lower than previous reports. SHV in* P. aeruginosa *mostly are located in chromosome and can play a hidden reservoir for these enzymes. Furthermore, the isolates harboring SHV-type genes are scarcely reported.^21^

**Table I:** Demographics of patients at both the hospitals

*Characteristics*	*No. (%)*
Mean ± standard deviation age (yr)	43.39±3.70
Gender	
Male	84 (68.29)
Female	39 (31.71)
Underlying disease	
Yes	18 (14.63)
No	105 (85.37)
Primary site of infection	
Blood	12 (9.76)
Brain shunt	1 (0.81)
Lung	37 (30.08)
Other	2 (1.63)
Swab	4 (3.25)
Tissue	1 (0.81)
Urine	57 (46.34)
Wound	9 (7.32)

**Table-II:** Multiple drug resistance patterns of the *P. aeroginusa* isolates at both the hospitals for SHV gene positive and negative

*Multiple drug resistance patterns*	*SHV*		
	*Positive*	*Negative*	*Total*
CRO+CAZ+TE+NOR+CTX+GM+CP+IMP+FEP+NA+CT	3.85%	7.69%	11.54%
CAZ+TE+NOR+CTX+GM+CP+IMP+FEP+NA+CT	3.85%	7.69%	11.54%
TE+NOR+CTX+GM+CP+IMP+FEP+NA+CT	3.85%	7.69%	11.54%
NOR+CTX+GM+CP+IMP+FEP+NA+CT	3.85%	7.69%	11.54%
CTX+GM+CP+IMP+FEP+NA+CT	3.85%	7.69%	11.54%
GM+CP+IMP+FEP+NA+CT	7.69%	7.69%	15.38%
CP+IMP+FEP+NA+CT	7.69%	7.69%	15.38%
IMP +FEP N+A+ CT	7.69%	7.69%	15.38%
FEP+NA+CT	11.54%	42.31%	53.85%
NA+CT	11.54%	46.15%	57.69%
CT	11.54%	69.23%	80.77%
TOTAL	15.38%	84.6%	100%

CP(ciprofloxacin), GM(gentamicin), TE(tetracycline), IPM(imipenem), NA(nalidiciciacid),

NOR(norfloxacine), FEP(cfefepim), CRO(cefdinir), CT(ceftizoxime), CTX(cefotaxime), CAZ(ceftazidime)

**Table-III:** Risk factors for acquisition of SHV type’s enzyme in P. aeruginosa strains isolated from 123 patients

*Variables*	*SHV-positive* *(13 patients),* *No. (%)*	*SHV-negative* *(110 patients),* *No. (%)*	*OR (95% CI)*	*P value*
Mean ± SD age (yr)	44.92	43.06	1.85 (-9.9-13.34)	0.749^ns^
Use of any antibiotics within previous two weeks	8 (6.6)	11 (9)	5.51 (1.85-16.43)	0.003^**^
Gender, male	12 (9.8)	65 (53.3)	2.09 (0.64-6.72)	0.161^ns^
Ventilator use	9 (7.4)	19 (15.6)	3.57 (1.29-9.83)	0.015^*^
Presence of catheter	10 (8.2)	22 (18)	3.63 (1.34-9.84)	0.011^*^
ICU hospitalization	13 (10.7)	36 (29.5)	3.40 (1.24-9.29)	0.014^*^
Trauma	9 (7.4)	33 (27)	1.71 (0.64-4.53)	0.202^ns^
Nosocomial infection	12 (9.8)	42 (34.4)	2.14 (0.80-5.69)	0.097^ns^
Blood transfusion	10 (8.2)	44 (36.1)	1.31 (0.5o-3.44)	0.373^ns^
Days of hospitalization	20	102	14.34 (2.87-25.8)	0.003^**^

*Significant at the 0.05 level

**Significant at the 0.01 level

ns: not significant

Multidrug Resistance (MDR) in Pseudomonas is different from throughout the world. In our study the rate of MDR isolate was 3.85% that lower than Turkey and Iran^11^^,^^22^ and higher than Europe and some American countries.^23^

## CONCLUSION

In this study prevalence of ESBL in pseudomonas infection has decreased in comparison with other studies in Iran. Blind therapy the *P. aeruginosa* infection without information from antimicrobial resistant may lead to increasing in risk factors, long hospitalization, persistence of infection and mortality rate. Appropriate infection control can prevent spread and outbreaks of ESBL-producing and MDR *P. aeruginosa.*
